# Accessory spinal nerve damage during a cervical lymph node biopsy: case report

**DOI:** 10.11604/pamj.2020.36.378.25292

**Published:** 2020-08-31

**Authors:** Hafid Arabi, Aziz Ahizoune, Rachid Benchanna, Nabil Abida, Salah Belasri, Badr Slioui, Amine Benjelloun

**Affiliations:** 1 Physical Medicine and Rehabilitation Unit, Avicenne Military Hospital, Marrakech, Morocco,; 2 Neurology Unit, Avicenne Military Hospital, Marrakech, Morocco,; 3 Pulmonology Unit, Avicenne Military Hospital, Marrakech, Morocco,; 4 Radiology Unit, Avicenne Military Hospital, Marrakech, Morocco

**Keywords:** Accessory spinal nerve, trapezius denervation, shoulder elevation deficit

## Abstract

The lesion of the accessory spinal nerve is often of iatrogenic origin. We report the case of an injury after a right jugulocarotid lymph node biopsy. A 30-year-old patient was referred for the treatment of right cervical lymphadenopathy suspected of tuberculosis. After the intervention and confirmation of tuberculosis diagnosis, the patient presented a functional impotence of the right shoulder and swarming of the right hand. The clinical examination found an active limitation of the shoulder, and a wasting of the upper bundle of the right trapezius muscle and the sternocleidomastoid. The EMG showed axonotmesis of the accessory spinal nerve and the MRI an amyotrophy of the trapezius with denervation edema. A simple rehabilitation has been scheduled. Damage of the accessory spinal nerve most often occurs after local surgery. EMG is essential for diagnosis. Rehabilitation is the first therapeutic option. Surgery can be considered if it fails. The surgeons must consider the protection of the accessory spinal nerve in case of cervical lymph node surgery.

## Introduction

The lesion of the accessory spinal nerve (ASN) is most often of iatrogenic origin [1]. It is responsible for a pure motor mononeuropathy. We report the case of an injury of the ASN after a cervical lymph node biopsy.

## Patient and observation

**Figure 1:**
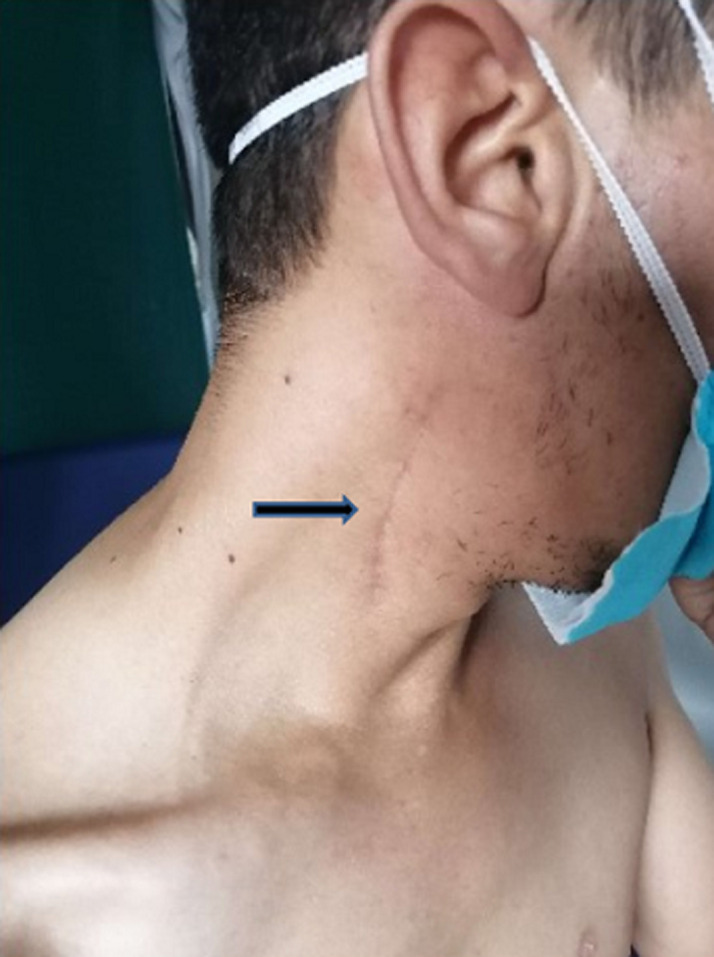
anterolateral scar on the right side of the neck

**Figure 2:**
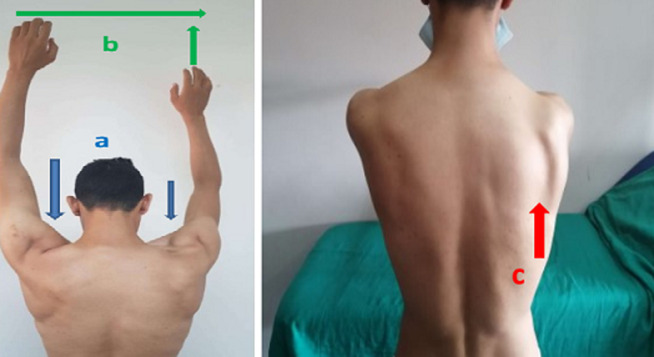
wasting of the upper bundle of the right trapezius muscle (A); limitation of the right shoulder elevation compared to the opposite side (B); slight detachment of the scapula (C)

**Figure 3:**
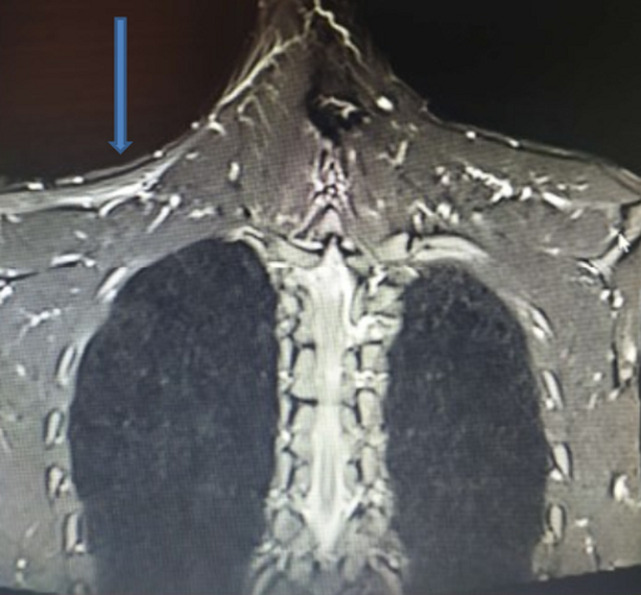
MRI of the brachial plexus, coronal cut in T2 sequence: wasting of the right trapezius muscle with denervation edema

A 30-year-old patient, with no previous medical history was referred for a right-sided jugular-carotid lymphadenopathy, without fever or deterioration in general condition. A biopsy was performed in ENT unit confirming the diagnosis of lymph node tuberculosis. There were no other locations of tuberculosis A 6 months anti-tuberculosis treatment has, therefore, been started. In the postoperative period, the patient began to complain of progressive partial functional impotence in the right shoulder accompanied by swarming in the right hand. The clinical examination showed an anterolateral clean scar on the right side of the neck (Figure 1), fall of the stump of the shoulder, a muscle wasting of the trapezius muscle and sternocleidomastoid, and a slight detachment of the scapula (Figure 2). The active elevation of the right shoulder was limited compared to the opposite side (Figure 2). The pinching of the right trapezius caused slight pain. The rest of the neurological examination of the brachial plexus was unremarkable. The global physical examination was normal. The electromyogram (EMG) study showed a scattered motor response of the right ASN with amplitude collapse on simulation above the lymph node biopsy wound. The study of the right trapezius muscle showed a rich spontaneous activity rich in its upper portion with a poor temporal summation in effort. There were no other anomalies in the territories of the collateral branches and terminal nerves of the brachial plexus. The long thoracic nerve was normal. The EMG concluded to an axonotmesis of the right accessory spinal nerve. A brachial plexus MRI was performed showing wasting of the right trapezius muscle with denervation edema (Figure 3). Functional rehabilitation has, therefore, been scheduled. Surgery will be considered in case of failure.

## Discussion

Mononeuropathy of the accessory spinal nerve is a rare iatrogenic motor disorder [1]. Idiopathic paralysis of this nerve has been described [2-4]. It affects the upper bundle of the trapezius and sternocleidomastodian muscle. The lesion generates a muscular weakness of these two muscles causing a deficit of elevation of the shoulder and the lateral rotation of the cervical spine without repercussions on the rest of the member musculature. However, we would like to point out that this slight detachment of the scapula in our patient is secondary to the weakness of the stabilizing muscles of the scapula (the anterior serrated muscle, the rhomboid and the angular). This detachment is a consequence of the non use of the limb and functional muscular overload on this musculature. This slight detachment of the scapula should not be attributed to a damage affecting the long thoracic nerve. This damage constitutes the differential diagnosis of the ASN lesion. The swarming sensation reported by our patient may be due to irritation of the brachial plexus during movement. Shoulder drop and muscle imbalance in the shoulder may be exacerbated by excessive stretching of the plexus [3]. The edema found on the MRI probably explains the slight pain on pinching the trapezius. To regain a trapezian function, this patient will be referred to rehabilitation for a protocol to strengthen the stabilizing and elevating muscles of the scapula. In the event of manifest handicap, and of failure of the rehabilitation, a reparative surgery is possible with a neurotisation of the accessory spinal nerve. It must be performed by an experienced surgeon with a perfect understanding of the cervical and scapular anatomy. Several procedures can be followed: the use of bundles of the upper trunk of the brachial plexus [5, 6]; the use of the lateral pectoral nerve [7]; or the use of the nerve fascicles of the C7 nerve [8]. The best treatment is of course preventive, and surgeons must consider the protection of the accessory spinal nerve in case of cervical lymph node surgery.

## Conclusion

Mononeuropathy of the accessory spinal nerve is often an iatrogenic condition, but spontaneous rupture is not rare. It requires a careful clinical examination of the shoulder musculature to recognize it. The electromyogram is mandatory. Functional treatment is the main therapeutic option, surgery is possible in case of failure. The best treatment remains the prevention with a necessary protection of the ASN in the lymph node biopsies.
